# Automated identification of pneumonia in chest radiograph reports in critically ill patients

**DOI:** 10.1186/1472-6947-13-90

**Published:** 2013-08-15

**Authors:** Vincent Liu, Mark P Clark, Mark Mendoza, Ramin Saket, Marla N Gardner, Benjamin J Turk, Gabriel J Escobar

**Affiliations:** 1 Division of Research and Systems Research Initiative, Kaiser Permanente, 2000 Broadway, Webster Annex CA 94612 Oakland, Northern California; 2 Santa Clara Medical Center, Kaiser Permanente, Santa Clara, CA, Northern California; 3 Vallejo Medical Center, Kaiser Permanente, Vallejo, CA, Northern California; 4 Walnut Creek Medical Center, Kaiser Permanente, Oakland, CA, Northern California

**Keywords:** Pneumonia, Intensive care unit, Natural language processing, Chest imaging, Electronic tool

## Abstract

**Background:**

Prior studies demonstrate the suitability of natural language processing (NLP) for identifying pneumonia in chest radiograph (CXR) reports, however, few evaluate this approach in intensive care unit (ICU) patients.

**Methods:**

From a total of 194,615 ICU reports, we empirically developed a lexicon to categorize pneumonia-relevant terms and uncertainty profiles. We encoded lexicon items into unique queries within an NLP software application and designed an algorithm to assign automated interpretations (‘positive’, ‘possible’, or ‘negative’) based on each report’s query profile. We evaluated algorithm performance in a sample of 2,466 CXR reports interpreted by physician consensus and in two ICU patient subgroups including those admitted for pneumonia and for rheumatologic/endocrine diagnoses.

**Results:**

Most reports were deemed ‘negative’ (51.8%) by physician consensus. Many were ‘possible’ (41.7%); only 6.5% were ‘positive’ for pneumonia. The lexicon included 105 terms and uncertainty profiles that were encoded into 31 NLP queries. Queries identified 534,322 ‘hits’ in the full sample, with 2.7 ± 2.6 ‘hits’ per report. An algorithm, comprised of twenty rules and probability steps, assigned interpretations to reports based on query profiles. In the validation set, the algorithm had 92.7% sensitivity, 91.1% specificity, 93.3% positive predictive value, and 90.3% negative predictive value for differentiating ‘negative’ from ‘positive’/’possible’ reports. In the ICU subgroups, the algorithm also demonstrated good performance, misclassifying few reports (5.8%).

**Conclusions:**

Many CXR reports in ICU patients demonstrate frank uncertainty regarding a pneumonia diagnosis. This electronic tool demonstrates promise for assigning automated interpretations to CXR reports by leveraging both terms and uncertainty profiles.

## Background

Pneumonia is a common cause of hospitalization [1,2]. In the intensive care unit (ICU), community- and hospital-acquired pneumonia are associated with substantial resource utilization, morbidity, and mortality [2,3]. Diagnosing pneumonia is often challenging since it requires both abnormal radiographic features and clinical findings [1,4]. In ICU patients, this diagnosis can be even more complex because of challenges in interpreting limited quality chest radiographs (CXRs) along with clinical data [2,4,5].

Prior studies demonstrate the suitability of natural language processing (NLP)—a methodology for encoding data from narrative reports—for assisting with automated pneumonia identification within CXR reports [6-12]. While these techniques are promising, few studies have addressed the question of whether they perform accurately in the ICU [13]. Given the complexity of identifying pneumonia in ICU CXRs, little is known about the additional relevance of ‘uncertainty’ in the language used by interpreting radiologists [4].

In this study, we evaluate 194,615 CXR reports from patients in the ICU. In a manually reviewed sub-sample, we describe how pneumonia-related and uncertainty terms influence report interpretation. We then describe an electronic tool, comprised of NLP queries and an algorithm to evaluate query profiles, that assigns automated determinations (‘positive’, ‘possible’, and ‘negative’) to reports. Finally, we evaluate its performance in a sample of reports drawn from ICU patient subgroups.

## Methods

### Setting and participants

The Kaiser Permanente Northern California (KPNC) Institutional Review Board approved this study. We conducted a retrospective analysis of CXR narrative reports from adult patients (age ≥ 18 years) with ICU admissions at 21 KPNC hospitals between October 2007 and December 2010. All hospitals used the same electronic health information systems providing centralized access to clinical and radiographic data [14-18]. For study patients, we collected data from all CXR reports completed during a single hospitalization.

Our analysis included the development of (1) a pneumonia lexicon; (2) a set of NLP queries to identify lexicon terms within reports; and (3) an electronic algorithm that used query results to provide CXR report interpretation. The performance of these tools was measured in a validation set of CXR reports as well as in a set of reports from two patient subgroups.

### Lexicon development

Two physicians experienced with critical care reviewed >1,000 CXR reports to empirically develop a lexicon focused on categorizing features associated with pneumonia (Table 1) within three broad categories: (1) terms and term groups; (2) uncertainty profiles; and (3) ‘other’ features. Terms and term groups were broadly divided based on whether or not they would be seen in pneumonia. For example, pneumonia terms included those considered equivalent to pneumonia or likely to represent pneumonia (pneumonia-equivalent, e.g., *bronchopneumonia* or *consolidation*) as well as those used to convey a pneumonia diagnosis in the correct context (pneumonia-related, e.g., *infiltrate* or *opacity*). Non-pneumonia terms included those related to alternate processes (e.g., *edema, atelectasis*) or those conveying negative or unrelated findings (‘*no acute cardiopulmonary disease’*).

**Table 1:** Development lexicon entries for terms and term groups and uncertainty profiles

**Terms and term groups**		**Uncertainty profiles**	
*Pneumonia-related*	*Non-pneumonia*	*Low uncertainty*	*High uncertainty or Versus*
Pneumonia	Atelectasis	Probable	Cannot exclude
Bronchopneumonia	Edema	Consider	Clinical correlation
Air bronchogram	Congestive heart failure	Concerning for	Could represent
Consolidation	Heart failure	Consistent with	Possible
Infiltrate	ARDS	Suspicious	Rule out
Opacity	Fluid overload	Suspect	Questionable
Density	Infarct	Suggestive of	Might
Pneumonitis	Contusion	Likely representing	May
Pneumonic	Hemorrhage	Compatible with	
Abcess	Mass		Versus
Aspiration	Low lung volume		Plus minus
Cavity	Hypoinflation		Or
Airspace disease/process	Congestion		And/or
Parenchymal process	Malignancy		/
	Nodule		
	Neoplasm		
	Collapse		
	Effusion		
	Scar		
	Fluid		

The table does not include all sub-combinations (‘pneumonic infiltrate’) or morphological variants (‘clinical correlation’ and ‘clinically correlate’).

Uncertainty profiles were classified as having versus phrasing (‘*pneumonia versus atelectasis’* or *‘consolidation/effusion’)*, low uncertainty (*‘probable pneumonia’*), or high uncertainty (*‘cannot exclude infiltrate’* ; Table 1). Based on these elements, individual pneumonia terms (*opacity*) could be linked with uncertainty profiles (e.g., *‘cannot exclude retrocardiac opacification’*). The lexicon also encoded ‘other’ features relevant to interpreting radiograph reports including those assessing disease progression (‘*worsening of infiltrates’*), anatomic location (*‘bilateral opacities’*), or stability (‘*unchanged from prior’* ).

### Natural language processing queries

Based on this lexicon, we developed a set of query strategies to flag the presence of terms and phrases within CXR reports (‘hits’) using an NLP-based software package that enables semantic information extraction from large document collections (I2E, Linguamatics [http://www.linguamatics.com]; United Kingdom). We applied these queries to CXR reports using the I2E software to count the number of query hits within individual reports. Each query was designed to capture a combination of the terms, features, and uncertainty profiles defined by the lexicon. For example, a frequent uncertainty construct used by interpreting radiologists juxtaposes pneumonia with an alternate diagnosis (e.g., ‘*pneumonia and/or atelectasis’*). Thus, our corresponding query (termed ‘pneumonia versus’) would generate two hits for the phrases ‘atelectasis versus bronchopneumonia’ and ‘edema/pneumonia’ within a single report. Queries were developed to incorporate focused negation so the phrases ‘without evidence of edema and/or pneumonia’ or ‘no atelectasis/pneumonia’ would not generate hits, while the phrase ‘no change in atelectasis versus pneumonia’ would. Similar ‘versus’ queries were also designed to identify other pneumonia-related term groups (e.g., ‘consolidation versus’, ‘infiltrate + versus’, ‘infection + versus’).

### Physician interpretation

To develop and validate our electronic algorithm for interpreting reports, we generated three sets of physician-interpreted CXR reports (development, derivation, validation). For each report, two physicians experienced with interpreting ICU CXR reports reached a consensus on whether the report was ‘positive’, ‘possible’, or ‘negative’ for pneumonia in a presumed scenario where CXRs were performed in patients whose clinical differential diagnosis included pneumonia (e.g., a patient with dyspnea). In the development (n = 777) and derivation (n = 950) sets, the physicians who created the lexicon and NLP queries assigned interpretations to randomly selected CXR reports. In the validation set, two other physicians (a radiologist and a pulmonary/critical care specialist) interpreted 739 additional CXR reports. The validation physicians had no role in the lexicon, query, and algorithm development; they were also blinded to the query and algorithm strategies.

### Electronic interpretation

Using the gold-standard physician interpretations in the development and derivation sets, we then developed an electronic algorithm for assigning interpretations to CXR reports. The algorithm included twenty steps where each step incorporated rules- or probability-based strategies to analyze combinations of NLP query hits (Table 2). For example, a CXR report that included a ‘blanket normal’ statement (e.g., *‘no acute cardiopulmonary findings’ *) without any other pneumonia terms would be assigned a ‘negative’ interpretation. A report that included only pneumonia terms within high uncertainty profiles (*‘infiltrate versus atelectasis’*) would be assigned a ‘possible’ interpretation.

**Table 2:** Overview of electronic algorithm steps used to interpret chest radiograph reports based on rules- and probability-based strategies

**Group (Step)**	**Determination**	**Rules**	**Predicted probability**
**Group 1** (Step 1)	Negative	‘Blanket Negative’ statement without any pneumonia-related terms	
**Group 1** (Step 2)	Negative	No pneumonia-related terms	
**Group 1** (Step 3)	Possible	High uncertainty pneumonia-related terms, no ‘blanket negative’ statement	
**Group 1** (Step 4)	Positive	Low/No uncertainty pneumonia-equivalent terms, no high uncertainty pneumonia-related terms, no non-pneumonia terms	
**Group 2** (Step 5)	Possible	High uncertainty pneumonia-related terms, no low/no uncertainty pneumonia-equivalent terms, no normal statement	
**Group 2** (Step 6)	Possible	Infiltrate + pneumonia-related terms, no low/no uncertainty pneumonia-equivalent terms	
**Group 2** (Step 7)	Possible	Any pneumonia-related versus terms	
**Group 3** (Step 8)	Positive	Low/no uncertainty pneumonia-equivalent terms, no blanket normal statement	
**Group 3** (Step 9)	Possible	Any uncertainty pneumonia-related terms	
**Group 3** (Step 10)	Possible	Any infiltrate + pneumonia-related terms, no non-pneumonia terms	
**Group 4** (Step 11)	Positive		Positive > 70%
**Group 4** (Step 12)	Negative		Negative >30%, Possible < 30%, Positive < 10%
**Group 4** (Step 13)	Possible		Possible > 10%, Negative < 10%, Positive < 10%
**Group 4** (Step 14)	Possible		Possible > 60%, Negative < 40%
**Group 4** (Step 15)	Positive	Any pneumonia-equivalent term	
**Group 4** (Step 16)	Possible		Possible > 20%, Positive > 10%
**Group 4** (Step 17)	Possible	Any uncertainty pneumonia-related terms, no low/no uncertainty pneumonia-equivalent terms	Negative < 30%
**Group 4** (Step 18)	Possible	Pneumonia-related terms, no non-pneumonia terms, no blanket normal statement	Negative < 40%
**Group 4** (Step 19)	Negative	Non-pneumonia terms	
**Group 4** (Step 20)	Possible	All remaining reports	

Reports that are assigned an interpretation based on a step are then removed from interpretation in the subsequent steps.

Because many reports included hits from several query elements that precluded simple rules-based interpretation, we also incorporated a set of predicted probabilities in selected algorithm steps. Using the development and derivation sets, we generated three logistic regression models to assign predicted probabilities that each report would have a ‘positive’, ‘possible’, or ‘negative’ interpretation. These probabilities were generated using backward stepwise logistic regression where NLP query hits associated with the binary outcome (e.g., for the ‘negative only’ outcome, negative = 1 and positive or possible = 0) with a p-value <0.2 were retained in the final model. The beta-coefficients, based on the derivation sample, were then used to calculate probabilities in the validation sample (Additional file 1). These probabilities were then used in concert with NLP query profiles to assign interpretations to reports that could not be classified simply with rules-based approaches. For example, after removing reports interpreted in the prior 11 steps, step 12 deemed a report ‘negative’ if its ‘negative’ predicted probability was >30%, its ‘possible’ probability was <30%, and its ‘positive’ probability was <10%.

### Algorithm performance

We evaluated algorithm performance in the validation set based on sensitivity, specificity, positive predictive values, and negative predictive values. To collapse the outcome into binary values, these were calculated for ‘Negative Alone’ (where negative reports were distinguished from either positive or possible), ‘Positive Alone’ (positive reports versus negative or possible reports), and ‘Possible Alone’ (possible reports versus negative or positive reports) categories. We also evaluated cumulative test characteristics based on grouped algorithm steps to determine their impact on performance.

Finally, we evaluated the accuracy of the algorithm in two ICU subgroups expected to have a high percentage of either negative or positive/possible CXR reports—patients admitted with pneumonia (n = 1,766) and with primarily rheumatologic or endocrine diagnoses (n = 1,201), as defined by Agency for Healthcare Research and Quality Clinical Classification Software codes (Additional file 1: Table S1) [19,20]. For both cohorts, we manually reviewed all ‘unexpected’ automated interpretation results (e.g., in the pneumonia cohort, a ‘negative’ CXR report within 48 hours of hospitalization would be an ‘unexpected’ finding) to assess whether the automated interpretations were accurate and categorize the report findings.

Analyses were conducted in Stata/SE 11.2 (College Station, TX). Results are reported as number (frequency) and mean ± standard deviation.

## Results

Study CXRs were randomly drawn from a total sample of 194,615 reports in 35,314 unique patients and 41,891 ICU admissions. Mean patient age was 65 ± 17 years; 52.6% of patients were male. Mean hospital length of stay was 8.8 ± 13.8 days. The mean number of CXR reports per patient was 4.2 ± 6.4.

### Physician interpretation

Two physicians manually interpreted 2,466 CXR reports by consensus; Table 3 shows examples of reports and physician-based interpretations from the validation set. In general, reports suggestive of pneumonia but whose findings could be seen in non-pneumonia conditions or required clinical data unavailable within the report were termed ‘possible’. ‘Negative’ reports were not suggestive of pneumonia, however, they could be consistent with other conditions like congestive heart failure. Of all physician-reviewed reports, most were deemed ‘negative’ (Table 4; range, 47.0% to 57.4%). A sizable fraction of reports were deemed ‘possible’ (overall, 41.7%) while only a small fraction were felt to be conclusively ‘positive’ (overall, 6.5%; validation, 7.2%).

**Table 3:** Selected examples of chest radiograph report determinations by category

**Positive**	
**1**	*There is new bilateral lower lobe consolidation with air bronchograms. There is some volume loss. Bibasilar pneumonias.*
**2**	*Again noted is the focal consolidation at the right lung base. It is not significantly changed and most likely represents middle lobe pneumonia. Right middle lobe air space opacity is probably pneumonia and not significantly changed.*
**Possible**	
**3**	*Interval clearing of the diffuse opacities of the lungs with residual opacities, findings suggesting alveolar edema, less likely pneumonia.*
**4**	*Endotracheal tube pulled back. Persistent cardiomegaly with congestive heart failure and bilateral pleural effusions. Bibasilar pneumonia is not excluded.*
**Negative**	
**5**	*Lungs are clear without pulmonary edema, focal consolidation, or pleural effusion. No acute cardiopulmonary disease.*
**6**	*Again seen are diffuse airspace opacities throughout both lungs, improved compared with the most recent prior examination. The pleural effusions appear smaller as well. Persistent pulmonary edema though it appears improved.*

**Table 4:** Frequency of clinician interpretation for radiographs by sample

		**Clinician interpretation, no. (%)**		
**Sample**	***n***	**Negative**	**Possible**	**Positive**
Blinded validation	*739*	424 (57.4)	262 (35.5)	53 (7.2)
Derivation	*950*	488 (51.4)	417 (43.9)	45 (4.7)
Developmental	*777*	365 (47.0)	350 (45.0)	62 (8.0)
Overall	*2,466*	1,277 (51.8)	1,029 (41.7)	160 (6.5)

### Lexicon and query development

The final lexicon included 52 terms/term groups, 27 uncertainty profiles, and 25 other terms/phrases not including morphological variants (e.g., *infiltrate, infiltration,* and *infiltrative*; (Table 1). In the final development stage, lexicon items, combinations, and uncertainty profiles were encoded into 31 unique I2E NLP queries. Nine queries flagged high uncertainty pneumonia features (to identify phrases like *‘infiltrate or edema’*, *‘pneumonia versus atelectasis*’), nine flagged low uncertainty pneumonia features (e.g., *‘probable pneumonia’, ‘suggestive of infiltrates’*), five flagged non-pneumonia features (e.g., *‘atelectasis’*, *‘pleural effusion’*), and eight flagged ‘other’ features (e.g., bilateral/multilobar location, new/progressive disease).

### I2E queries

When applied to the total sample of 194,615 CXR reports, the 31 I2E queries produced a total of 534,322 hits. The mean number of hits per report was 2.7± 2.6, ranging from zero to 38. Additional file 1: Figure S1 shows a schematic example of the variety of query hits that would be identified in a CXR report interpreted as ‘possible’ pneumonia. In the validation set, the queries identified a total of 2,228 hits, including 806 (36.2%) for ‘other’, 638 (28.6%) for non-pneumonia, 547 (24.6%) for low uncertainty pneumonia, and 237 (10.6%) for high uncertainty pneumonia features.

### Electronic algorithm

The final electronic interpretation algorithm—based on testing in the development and derivation cohorts—was divided into 4 groups comprised of 20 steps (Table 2). The first 3 groups, including 10 steps, were entirely rules-based; the 10 steps in the final group combined rules and predicted probabilities. For example, the first step in the algorithm encoded all CXR reports with a negative/normal phrase (e.g., *‘no acute cardiopulmonary disease’*) and without any pneumonia-relevant terms as ‘negative’. The third step encoded reports containing only low or no uncertainty pneumonia-equivalent phrases as ‘positive’. Step 18, including both rules and probabilistic approaches, encoded reports as ‘possible’ if they included high uncertainty pneumonia-related terms and had a predicted probability of being negative of <30%. Table 5 shows the test characteristics of the algorithm in the derivation set.

**Table 5:** Test characteristics of the automated interpretation algorithm by sample

	**Test characteristics by interpretation samples (%)**			
**Dataset**	***Sensitivity***	***Specificity***	***PPV***	***NPV***
	**Negative-only (versus Positive or Possible)**			
Validation	92.7	91.1	93.3	90.3
Derivation	93.2	96.8	96.8	93.1
Overall	92.8	93.1	93.5	92.3
	**Positive-only (versus Possible or Negative)**			
Validation	45.3	99.0	77.4	95.9
Derivation	53.3	99.0	72.7	97.7
Overall	45.0	99.0	75.8	96.3
	**Possible-only (versus Positive or Negative)**			
Validation	86.6	87.4	79.1	92.3
Derivation	94.2	89.9	87.9	95.2
Overall	89.9	87.5	83.8	92.4

*PPV* Positive predictive value, *NPV* Negative predictive value.

### Validation set performance

In the validation set, the performance of the algorithm was in a lower, but similar, range to that in the derivation set (Table 5). For the ‘Negative Alone’ category, the sensitivity was 92.7%, specificity 91.1%, positive predictive value 93.3%, and negative predictive value 90.3%. For the ‘Positive Alone’ category, the sensitivity (45.3%) and positive predictive value (77.4%) were substantially lower. For the ‘Possible Alone’ category, test characteristics ranged from 79.1% (positive predictive value) to 92.3% (negative predictive value). Most CXR reports (70.2%) could be categorized within the algorithm’s first four steps (Additional file 1: Table S2). Those that could not be categorized by query rules alone—19.2% of the total sample (group 4)—were associated with worsened test characteristics.

### ICU sub-samples

Among CXR reports in the ICU pneumonia cohort, the electronic algorithm interpreted 1,249 (70.7%) as possible, 360 (20.4%) as positive, and 157 (8.9%) as negative. A manual review of the 157 unexpected ‘negative’ reports demonstrated that the algorithm misclassified seven reports (4.5%; Table 6). The remaining reports were correctly interpreted and were either normal (31.8%) or included radiologist interpretations consistent with non-pneumonia conditions (e.g., heart failure, 21.7%). Among CXR reports for patients admitted with endocrine or rheumatologic diagnoses, the algorithm incorrectly interpreted 10 (7.1%) reports. The remaining reports were suggestive of pneumonia or specifically communicated uncertainty about the diagnosis (Table 6).

**Table 6:** Audit results of ‘unexpected’ chest radiograph results among ICU patients with pneumonia and endocrine/rheumatologic diagnoses

**Results by ICU admission diagnosis class**			
**Pneumonia**		**Endocrine/Rheumatologic**	
Category	Number (%)	Category	Number (%)
*Incorrect reading*	*7 (4.5)*	*Incorrect reading*	10 (7.1)
Normal report	50 (31.8)	Pneumonia-relevant term	65 (46.1)
Heart failure	34 (21.7)	Atelectasis versus pneumonia-relevant	40 (28.4)
Other (e.g., mass, nodules)	27 (17.2)	Edema versus pneumonia-relevant	11 (7.8)
Atelectasis	16 (6.4)	Other	8 (5.7)
Hypoinflation	10 (4.5)	Pneumonia	7 (5.0)
Interstitial markings	5 (3.2)		
Diaphragmatic process	4 (2.5)		
Scar/chronic process	4 (2.5)		

## Discussion

In this study, we evaluated a large sample of chest radiograph reports from critically ill patients. Among nearly 2,500 reports categorized by manual review and physician consensus, 42% could not be classified as either ‘negative’ or ‘positive’. In many cases, these ‘possible’ reports included language from interpreting radiologists that conveyed frank uncertainty about whether the findings represented pneumonia or another condition with an appearance similar to pneumonia. In these cases, interpreting physicians felt that additional clinical information, beyond the CXR report, were necessary to determine whether a pneumonia was present or absent. Only a minority of reports (6.5%) included language that was deemed conclusive for, or highly likely to be, pneumonia.

In light of these challenges in categorizing ICU CXR reports into traditional ‘negative’ or ‘positive’ bins, we designed an algorithm that leveraged the wide range of uncertainty conveyed by radiologists. While this tool incorporated a set of complex techniques, the time required to analyze nearly 200,000 CXR reports—the estimated number of reports that would be generated at our 21 ICUs over 2 years—was as low as 10 minutes after document indexing. This electronic tool demonstrated very good performance in identifying ‘negative’ CXR reports. It also had high specificity for identifying ‘positive’ CXRs but had lower sensitivity and positive predictive value. Finally, it demonstrated good performance in identifying the sizable number of ‘possible’ CXR reports, a category that has not been well characterized in prior studies.

Pneumonia is a common and costly cause of hospitalization and is associated with substantial morbidity and mortality [1,2]. Among critically ill patients, hospital-acquired or ventilator-associated pneumonia further contribute to significant increases in length of stay, hospital costs, and mortality [2,3]. Prior studies have found that electronic tools can accurately identify abnormal radiograph reports and, thus, have the potential to improve clinical decision making and bedside care, quality and performance improvement, and adverse event or outcomes reporting [6-13,21-25]. Furthermore, when deployed on a large scale, these tools can be applied at a relatively low cost when compared with manual chart review. However, the interpretation tools in prior studies often considered CXR reports as a binary variable (negative/positive), limiting their diagnostic utility, especially in complex ICU patients [4].

A recent study by Dublin and others evaluated the performance of an open-source NLP system (ONYX) to assist with differentiating electronic CXR reports that required further manual review from those that could be conclusively labeled as ‘consistent’ or ‘inconsistent’ with pneumonia [26]. Out of 5,000 reports, between 12% and 25% were determined as requiring additional manual review—a lower, but still substantial, number of reports compared with our study. In their study, some criteria used to determine which reports required manual review were similar to those in our study (e.g., the presence of both atelectasis and pneumonia). In the remaining reports, their NLP system demonstrated excellent test characteristics similar to, or better than, those reported in prior NLP CXR report studies [6,8,9,26,27]. It is important to note the substantial differences in the patient populations from which the CXR reports were obtained. In the Dublin study, for example, 92% of reports were from outpatients—a population in whom radiographic image quality is expected to be higher and features like atelectasis or infiltrates are expected to be less prevalent [26].

Among inpatients, a new or progressive radiographic abnormality is necessary to raise the suspicion of pneumonia, however, the final diagnosis depends on a constellation of other clinical features (e.g., vital signs, symptoms, history, microbiology) [1,2]. In the ICU, diagnosing pneumonia is even more difficult because of technical challenges related to interpreting portable CXRs in supine patients with catheters, ventilators, devices, or competing conditions that can mimic pneumonia (e.g., fluid overload, atelectasis, lung hypo-inflation) [4,5]. Furthermore, in the ICU, the diagnosis of pneumonia can sometimes only be confirmed after treatment is administered and a patient’s response is ascertained [2]. Our tool, which was built with these challenges in mind, helps extend the capabilities of prior NLP-based approaches that largely relied on a more proscribed set of terms without evaluating the significant uncertainty communicated by radiologists [6,7,9,10,13].

Prior NLP studies have also evaluated the role of uncertainty in accurately interpreting biomedical reports [28-30]. For example, Vincze and others describe the development of the BioScope corpus which is annotated for a wide range of negations and linguistic speculations [28]. Many of the uncertainty profiles we captured in our lexicon are also described by the BioScope investigators including syntactic structures that connote ambiguity through auxiliaries, adjectives, or adverbs that are associated with keywords of interest. While the BioScope corpus contains free text from a wide variety of sources, including medical texts, biologic manuscripts, and abstracts, our corpus is drawn from a relatively proscribed source with a set of common and well-defined terms and phrases. As a result, the uncertainty profiles used in our NLP queries may have limited applicability to other free text sources. For example, common uncertainty phrases in CXR reports like *‘cannot exclude infiltrates’* may be infrequent in routine scholarly manuscripts or medical texts.

While our tool performed well independently, we designed it so that it could be overlaid with other detailed clinical, physiologic, and treatment data; essentially, the same data that clinicians use to confirm pneumonia in patients with an abnormal radiograph [2]. Using these additional diagnosis data in two ICU patient subgroups, we found that the algorithm continued to demonstrate very good performance in accurately assigning CXR report interpretations. We are currently incorporating this tool within more complex database structures that include detailed data about vital signs, ventilator settings and duration, antibiotic administration, and culture results [18]. This set of tools could be useful in a variety of healthcare domains. For example, in our healthcare system, quality improvement efforts aim to reduce the frequency of healthcare- or ventilator-associated pneumonia, however, these efforts are limited by the resource strain of reviewing CXR reports among all hospitalized patients to identify relevant cases [2,31]. Our tool could be used to automatically evaluate all CXR reports in hospitalized patients and flag those whose cases require further detailed review. This tool could also be used in conjunction with electronic decision support tools that aid clinicians in correctly triaging pneumonia patients and choosing appropriate antibiotics [11,25,31,32]. Finally, as applied in the study by Dublin et al., these tools can aid in lowering the burden of chart review for research studies [26].

This study has several important limitations. First, while it included 21 hospitals, the CXR reports were all drawn from a single integrated healthcare delivery system in Northern California. It is possible that when applied to an external population of patients and interpreting radiologists, the performance of this algorithm might suffer because of differences in language across regions or institutions. Second, the queries were built within the proprietary I2E software package potentially presenting barriers to dissemination. However, we designed the query framework to be adaptable to other NLP-based search tools to foster future open-source availability. Finally, in this study, we developed these tools to analyze reports in a retrospective, rather than a real-time, setting. Our future development aims to provide real-time report indexing and querying to support the tool’s applications at the point of bedside care.

## Conclusions

More than 40 percent of chest radiograph reports from critically ill patients demonstrated uncertainty in assigning a diagnosis of pneumonia. An automated tool based on a set of natural-language processing-based queries and algorithms showed very good performance for accurately assigning ‘positive’, ‘possible’, and ‘negative’ determinations in these reports, both when tested independently and in patient subgroups. This electronic tool demonstrates promise for using large-scale automated detection of suspicious findings from chest radiographs for clinical, operational, and reporting efforts.

## Competing interests

The authors declare that they have no competing interests.

## Authors’ contributions

VL had full access to the data and takes responsibility for the integrity of the data and the accuracy of the data analysis. VL contributed to study design; data collection, analysis, and interpretation; preparation of the manuscript; and review and approval of the final manuscript. MC contributed to study design; data analysis and interpretation; preparation of the manuscript; and review and approval of the final manuscript. MM contributed to study design, data analysis, and preparation and approval of the final manuscript. RS contributed to study design, data analysis, and preparation and approval of the final manuscript. MG contributed to study design, data analysis and interpretation, and preparation and approval of the final manuscript. BT contributed to study design, data analysis, and preparation and approval of the final manuscript. GE contributed to study design, data analysis and interpretation, preparation of the manuscript, and review and approval of the final manuscript.

## Pre-publication history

The pre-publication history for this paper can be accessed here:

http://www.biomedcentral.com/1472-6947/13/90/prepub

## Supplementary Material

Additional file 1 Supplemental tables and figures for automated identification of pneumonia in chest radiograph reports in critically ill patients. Click here for file

## Prior abstract

Portions of this research were presented at the 2012 and 2013 American Thoracic Society conferences.
