# Supplementary material for: Automated identification of pneumonia in chest radiograph reports in critically ill patients
Source: BMC Med Inform Decis Mak. 2013 Aug 15;13:90. doi: 10.1186/1472-6947-13-90 (PMC3765332; doi:10.1186/1472-6947-13-90)
Supplement: Additional file 1 — Supplemental tables and figures for automated identification of pneumonia in chest radiograph reports in critically ill patients. [file 1472-6947-13-90-S1.doc]

**Supplemental Materials.**

**e-Figure 1. Sample query elements identified by I2E in radiograph report.**Features include pneumonia terms (black boxes), non-pneumonia terms (grey box), uncertainty features (grey shading), anatomical location (solid line), and temporal changes (dotted line).

**e-Table 1**. **AHRQ Clinical Classification Software codes for admitting diagnoses of ICU patient subgroups.** Admitting diagnoses were assigned based on physician-entered diagnoses at the time of hospital admission from the emergency department.

| **Diagnosis subgroup** | |  | | **AHRQ CCS code** | **Description** |
| --- | --- | --- | --- | --- | --- |
|  | |  | |  |  |
| **Pneumonia** | |  | | 122 | Pneumonia |
|  |  | | 129 | | Aspiration pneumonitis, food/vomit |
|  | |  | |  |  |
| **Endocrine/Rheumatologic** | |  | | 48 | Thyroid disease |
|  |  | | 49 | | Diabetes mellitus without complication |
|  |  | | 50 | | Diabetes mellitus with complication |
|  |  | | 51 | | Other endocrine disorders |
|  |  | | 53 | | Disorders of lipid metabolism |
|  |  | | 54 | | Gout and other crystal arthropathies |
|  |  | | 57 | | Immunity disorders |
|  |  | | 200 | | Other skin disorders |
|  |  | | 202 | | Rheumatoid arthritis and related diseases |
|  |  | | 210 | | Systemic lupus erythematosus |
|  |  | | 211 | | Other connective tissue disease |
|  | |  | |  |  |

| **e-Table 2. Test characteristics based on interpretation algorithm steps.** | | | | | | | | | | | | |
| --- | --- | --- | --- | --- | --- | --- | --- | --- | --- | --- | --- | --- |
|  |  | Determination Steps | | | **Cumulative Test Characteristics in Validation Sample** | | | | | | | |
|  |  |  |  |  | Negative alone | | | | Positive alone | | | |
| **Algorithm** | N (%) | Negative | Possible | Positive | Sens | Spec | PPV | NPV | Sens | Spec | PPV | NPV |
|  |  |  |  |  |  |  |  |  |  |  |  |  |
| **Group 1 (Rules)** | 519 (70.2) | 2 | 1 | 1 | 98.9 | 90.2 | 96.4 | 97.0 | 67.9 | 99.0 | 79.2 | 98.2 |
|  |  |  |  |  |  |  |  |  |  |  |  |  |
| **Group 2 (Rules)** | 56 (7.6) | 0 | 3 | 0 | 98.7 | 92.9 | 96.4 | 97.4 | 55.9 | 99.1 | 79.2 | 97.3 |
|  |  |  |  |  |  |  |  |  |  |  |  |  |
| **Group 3 (Rules)** | 31 (4.2) | 0 | 2 | 1 | 98.7 | 93.9 | 96.4 | 97.7 | 45.7 | 99.1 | 80.8 | 95.7 |
|  |  |  |  |  |  |  |  |  |  |  |  |  |
| **Group 4 (Rules + Probability)** | 142 (19.2) | 2 | 6 | 2 | 92.7 | 91.1 | 93.3 | 90.3 | 45.3 | 99.0 | 77.4 | 95.9 |
|  |  |  |  |  |  |  |  |  |  |  |  |  |

**e-Appendix 1. Logits by outcome type**(‘positive only’, ‘possible only’, ‘negative only’)**.** Coefficients rounded to one decimal place.**Abbreviations.** PNA: pneumonia; Versus: versus phrasing; HighPT: high pre-test probability; LowPT: low pre-test probability; NoU: no uncertainty

**‘Positive only’ logit** = -4.9 + (-4.5 * Normal) +

(-1.4 * PNA versus) + (9.2 * PNA HighPT) +

(-2.7 * PNA LowPT) + (3.4 * PNA NoU) +

(-2.6 * Action Required) + (1.0 * Stable) +

(2.8 * Consolidation-groupNoU) + (.9 * new PNA-equivalent) +

(1.0 * weakPNA-equivalentNoU) + (.5 * Infiltrate-groupNoU) +

(-1.3 * Non-PNANoU)

**‘Possible only’ logit** = -1.0 + (-1.6 * Normal) +

(4.0 * PNA versus) + (-4.3 * PNA HighPT) +

(3.7 * PNA LowPT) + (-.8 * PNA NoU) +

(3.8 * Consolidation-groupVersus) + (-.7 * Action Required) +

(2.4 * Infiltrate-groupHighPT) + (2.1 * Infiltrate-groupLowPT) +

(1.5 * Infiltrate-groupNoU) + (-.5 * Non-PNANoU) +

(.3 * Left-sided) + (-.3 * new PNA-equivalent) +

(1.2 * weakPNA-equivalentNoU) + (-1.1 * Non-PNANoU)

**’Negative only’ logit**= .9 + (1.5 * Normal) +

(-3.3 * PNA NoU) + (-2.7 * Consolidation-groupNoU) +

(-2.3 * Infiltrate-groupHighPT) + (-1.0 * Infiltrate-groupLowPT) +

(-1.9 * Infiltrate-groupNoU) + (.3 * Non-PNANoU) +

(-2.1 * weakPNA-equivalentVersus) + (-0.9 * weakPNA-equivalentNoU) (1.2 * Non-PNANoU) + (-1.0 * Multilobar) + (-.6 * Left-sided) +

(-.9 * Right-sided) + (1.5 * Action Required)
